# Multimodal *In Vivo* Imaging of Tumorigenesis and Response to Chemotherapy in a Transgenic Mouse Model of Mammary Cancer

**DOI:** 10.1007/s11307-015-0916-7

**Published:** 2015-12-02

**Authors:** Jean-Louis Alberini, Raphaël Boisgard, Stéphanie Guillermet, Karine Siquier, Benoît Jego, Benoît Thézé, Saik Urien, Keyvan Rezaï, Emmanuelle Menet, Renaud Maroy, Frédéric Dollé, Bertrand Kühnast, Bertrand Tavitian

**Affiliations:** CEA, DSV, I2BM, Service Hospitalier Frédéric Joliot, Laboratoire d’Imagerie Moléculaire Expérimentale, Orsay, France; Service de Médecine nucléaire, Institut Curie, Hôpital René Huguenin, Saint-Cloud, France; Faculté de Médecine, Université Versailles Saint-Quentin, Versailles, France; Service de pharmacologie, Institut Curie, Hôpital René Huguenin, Saint-Cloud, France; Service de pathologie, Institut Curie, Hôpital René Huguenin, Saint-Cloud, France; Université Paris Descartes Sorbonne Paris Cité, Assistance Publique-Hôpitaux de Paris, Hôpital Européen Georges Pompidou, Radiology Department, Paris, France; Université Paris Descartes Sorbonne Paris Cité, INSERM UMR 970, Cardiovascular Research Center - PARCC, 56 rue Leblanc, 75015 Paris, France

**Keywords:** Molecular imaging, Biomarker, Breast cancer, Response to treatment, Transgenic model, Sodium-iodide symporter

## Abstract

**Purpose:**

Transgenic mice expressing the polyoma middle T oncoprotein (PyMT) in the mammary epithelium were explored by multimodal imaging to monitor longitudinally spontaneous tumor growth and response to chemotherapy.

**Procedures:**

Positron emission tomography (PET) with 2-deoxy-2-[^18^F]fluoro-d-glucose ([^18^F]FDG) and 3'-deoxy-3'-[^18^F]fluorothymidine ([^18^F]FLT), single photon emission tomography (SPECT) with [^99m^Tc]TcO_4_ ([^99m^Tc]TEC), X-ray computed tomography, and fluorescent confocal endomicroscopy (FCE) images were acquired during tumor progression in female PyMT mice. Imaging with [^18^F]FDG and [^99m^Tc]TEC was also performed in untreated, doxorubicin-treated, and docetaxel-treated PyMT mice. Total tumor volumes were quantified. Tumors were collected and macroscopic and histological examinations were performed.

**Results:**

All PyMT mice developed multifocal tumors of the mammary epithelium that became palpable at 8 weeks of age (W8). Computed tomography (CT) detected tumors at W14, while a clear tumoral uptake of [^99m^Tc]TEC and [^18^F]FDG was present as early as W6 and W8, respectively. No contrast between mammary tumors and surrounding tissue was observed at any stage with [^18^F]FLT. FCE detected an angiogenic switch at W10. Lung metastases were not clearly evidenced by imaging. Doxorubicin and docetaxel treatments delayed tumor growth, as shown by [^18^F]FDG and [^99m^Tc]TEC, but tumor growth resumed upon treatment discontinuation. Tumor growth fitted an exponential model with time constant rates of 0.315, 0.145, and 0.212 week^−1^ in untreated, doxorubicin, and docetaxel groups, respectively.

**Conclusions:**

Molecular imaging of mammary tumors in PyMT is precocious, precise, and predictive. [^18^F]FDG-PET and [^99m^Tc]TEC SPECT monitor tumor response to chemotherapy.

**Electronic supplementary material:**

The online version of this article (doi:10.1007/s11307-015-0916-7) contains supplementary material, which is available to authorized users.

## Introduction

The ability of *in vivo* imaging to recognize tumors in a precocious, precise, and predictive manner improves detection, staging, and response to treatment of cancer. Animal models of cancer can be useful to test imaging methods for tumor progression and response to treatment in longitudinal studies. However, physiological differences between species in drug metabolism, circulation, plasmatic concentration of metabolites and nutriments, etc. may influence imaging results and may reduce the clinical interest of translational studies, especially in models where tumors grow in a xenogeneic environment and/or heterotopically. Transgenic models have the strengths that tumors develop *in situ* in the native tissue, although tumorigenesis is often a slow and stochastic process, which may render difficult and tedious the constitution of homogeneous groups of animals for comparison purposes.

The transgenic mouse model of mammary carcinoma caused by expression of the polyoma middle T oncoprotein (PyMT) in the mammary epithelium [1] mimics the Luminal B type of human breast cancer. Although it does not recapitulate all of the human disease (absence of bone and brain metastases, loss of hormone receptors during progression), it offers several advantages for imaging studies of breast carcinogenesis: (i) tumors develop rapidly (2–3 months) in the mammary glands localized along parasagittal lines in the neck, chest, abdomen, and pelvis parts, (ii) penetrance of the phenotype is ~100 % in heterozygous PyMT^+/−^ female mice, (iii) progression to malignancy offers similarities with that observed during human ductal carcinoma carcinogenesis [2], including frequent occurrence of lymph node and lung metastases at late stages [2], and (iv) PyMT tumors express many of the genes overexpressed in human luminal tumors, including estrogen and progesterone receptors (ER and PR positive) and the sodium-iodide symporter (NIS) [3]. In the present study, we applied *in vivo* imaging techniques for the assessment of early tumor response in female PyMT mice. We performed X-ray computed tomography (CT), positron emission tomography (PET) with 2-deoxy-2-[^18^F]fluoro-d-glucose ([^18^F]FDG) and 3'deoxy-3'-[^18^F]fluorothymidine ([^18^F]FLT), single photon emission computed tomography (SPECT) with [^99m^Tc]TcO_4_ ([^99m^Tc]TEC), and fluorescent confocal endomicroscopy (FCE) of microvessels in longitudinal weekly follow-up to detect mammary tumors and lung metastases [1]. *In vivo* imaging was repeated in groups of animals receiving standard chemotherapy for breast cancer: doxorubicin, an anthracyclin, or docetaxel, a taxane. *In vivo* imaging data were correlated with immunohistochemistry of Ki-67 and NIS expression.

## Material and Methods

### Animals

Animal experiments were approved by the local ethical committee and followed the ARRIVE guidelines of the National Centre for the Replacement, Refinement, and Reduction of Animals in Research (London, UK). Female heterozygous PyMT^−/+^ mice were obtained by crossing wild-type FVB females with heterozygous PyMT^−/+^ males. Offsprings were genotyped by qualitative PCR. ADN was extracted from 25 mm^3^ of mouse tail using 100 μL of Extract-N-Amp™ PCR ReadyMix™ incubated for 10 min at 25 °C followed by 3 min at 95 °C. Primers were PyMT3/PyMT and PO up/PO low for transgenic and FVB, respectively.

Animals were housed separately at constant temperature (21 °C) and relative humidity (60 %) under a regular light/dark schedule. Food and water were freely available. Mice were randomly assigned to the untreated or treated group. Mean total tumor volume (TTV) per mouse was defined as the sum of all tumors visible in the five pairs of mammary glands in a given mouse. Imaging was performed weekly in non-fasted mice anesthetized with 2 % isoflurane. A heat lamp was used to maintain body temperature. Animals were sacrificed under terminal anesthesia at 14 weeks of age (W14) in the untreated group and at W18 in the treated groups. In total, 96 animals were used for imaging and biological experimentations.

### Chemotherapy

#### Preliminary Toxicity Studies 

In order to determine the maximal tolerated dose (MTD), the cytotoxic drugs doxorubicin and docetaxel were administered to two groups of five FVB mice. Doxorubicin (Doxorubicin^®^, Teva Pharmaceuticals, France) diluted in 0.9 % NaCl was injected intraperitoneally (IP) in five mice at a dose of 10 mg/kg weekly during three successive weeks [4]. Docetaxel (Taxotere^®^, Sanofi-Aventis, France) was diluted in its specific excipient and given to two different groups of four mice either intravenously (IV) or IP at different doses (15, 20, and 30 mg/kg) in three successive administrations at 4-day intervals. After drug administration, all animals were monitored during 2 months by clinical examination, weight assessment every other day, and dental examination every week.

In the doxorubicin-treated group treated with a 10-mg/kg dose, a 20 % weight loss with a 15-day nadir was observed. No other signs of toxicity were observed, and this dose was deemed acceptable. In the group treated with 30 mg/kg docetaxel IV, a 20 % weight loss was observed, while in the group treated by 30 mg/kg docetaxel IP, weight loss was 10 % with a 12-day nadir. However, in the IV-treated group, in two cases, we observed severe muscular paralysis leading to muscular atrophy 2 weeks after the first injection, followed by partial recovery at 9 weeks. This side effect was not observed in the group treated by the IP route. Considering the possibility of supplementary toxicity due to anesthesia and in order to perform a longitudinal follow-up, in the present study, docetaxel was administered IP at 20 mg/kg.

#### Chemotherapy Protocol for Imaging

Homozygous PyMT mice from the same littermate were randomly assigned to one of the following groups: (i) sham-treated; (ii) three IP injections of doxorubicin, 10 mg/kg weekly from W9 to W11; and (iii) three IP injections of docetaxel, 20 mg/kg every 4 days from W9 to W10. These regimens improve chemosensitivity in mouse models of breast cancer ([4] and E. Marangoni and M.F. Poupon, personal communication).

Four mice from each group were imaged with PET from W8 to W18, and four mice from each group were imaged with SPECT from W8 to W18. The administration of cytotoxic drugs was always performed after the SPECT and PET imaging sessions. Body weights were recorded two to three times per week. No tooth loss was observed during the follow-up.

### Imaging

#### CT

 Whole body CT scans were acquired on the Fast micro-CT scanner SkyScan 1178 (Skyscan, Kontich, Belgium) operated at 50 keV and a rotational angle of 180° with averaging of 4 and respiratory gating. Pixel size was 160 μm, and the total scan duration was approximately 4 min. No contrast medium was administered.

### [^18^F]FDG-PET Imaging

#### Image Acquisition 

It has been reported that isoflurane anesthesia induces large variations of glycemia in fasted mice but is not a major confounding factor for reproducibility of [^18^F]FDG-PET [5]. Therefore, in the present study, [^18^F]FDG was administered in non-fasted animals and scanning protocols (time after injection and duration) were standardized as recommended.

A first group of PyMT mice (*n* = 4) was imaged weekly from W8 to W14 (except for 1 mouse that was also imaged until W16) after tail vein injection of 7 MBq (200 μCi) of [^18^F]FDG (Cisbio, Orsay, France) diluted in 100 μl of a saline solution. Under isoflurane anesthesia, serial dynamic PET images (60 s × 5, 120 s × 5, 300 s × 3, 600 s × 3, 900 s × 2) were acquired in a microPET Focus 220 (CTI-Concorde Microsystems Inc., USA) in 3D mode during 90 min, starting immediately after the injection of [^18^F]FDG. Time–activity curves showed that [^18^F]FDG uptake increased exponentially and reached a plateau approximately 50–60 min after injection.

#### Image Reconstruction and Analysis 

Images were reconstructed using OSEM 2D with four iterations and 16 subsets and with a correction attenuation map calculated from the first images [6]. Dynamic images were analyzed with Anatomist software (SHFJ/CEA, France) using semi-automatic segmentation software (local mean analysis method or LMA) [7]. The metabolic tumor volume (MTV) was assessed as the summed volume of all tumoral mammary glands showing higher [^18^F]FDG uptake than the surrounding tissue.

### [^18^F]FLT-PET Imaging

#### [^18^F]FLT Synthesis 

[^18^F]FLT was synthesized according to a procedure slightly modified from Yun *et al.* [8] and Oh *et al.* [9]. Briefly, fluorine-18, as dried K[^18^F]F-K222 complex, was introduced into the precursor 3-*N*-Boc-5'-*O*-dimethoxytrityl-3'-*O*-nosyl-thymidine (ABX, Germany), in 800 μL of acetonitrile at 165 °C for 1 min. After a rapid purification on a silica cartridge, the *O*-dimethoxytrityl and *N*-Boc protective groups were removed using aqueous HCl 4 N at 90 °C for 5 min. The final purification was performed on preparative reverse-phase HPLC, eluted with a mixture of aqueous 0.9 % NaCl and ethanol (92:8, *v*/*v*). [^18^F]FLT with chemical and radiochemical purities higher than 95 and 98 %, respectively, was obtained in a 15 to 20 % radiochemical yield (decay corrected) with a specific radioactivity ranging from 260 to 330 GBq/μmol (decay corrected).

#### Image Acquisition

 Dynamic PET acquisitions were acquired during 90 min (60 s × 5, 120 s × 5, 300 s × 3, 600 s × 3, 900 s × 2 images) in four PyMT mice with large tumors (W14) after tail vein injection of 7 MBq (200 μCi) of [^18^F]FLT (CEA, Orsay, France) diluted in 100 μl of saline solution.

### SPECT Imaging with [^99m^Tc]TEC

#### Image Acquisition 

SPECT images were performed on a one-head 10-cm field-of-view parallel-hole collimator gamma camera combined with a CT scanner (Gamma Imager, Biospace Lab, France), 15 min after tail vein injection of 7 MBq (200 μCi) of [^99m^Tc]TEC (Cisbio, Orsay, France) diluted in 100 μL of saline solution.

#### Image Analysis

 Images were reconstructed using an OSEM 2D protocol and analyzed with Amira ™ software (FEI, USA). Mammary tumors in PyMT mice showed a clear higher [^99m^Tc]TEC uptake than adjacent tissue. They were manually delineated, and their volume was measured and summed to yield TTV.

### *In Vivo* Fluorescence Imaging

*In vivo* fluorescence imaging was performed 15 min after intravenous injection of 200 μl Dextran-FITC (30 mg/ml), using a fibered FCE (Cellvizio^®^ Mauna Kea Technologies, France). The tip of the imaging probe was placed in contact with the abdominal mammary glands. FVB (*n* = 5) and untreated (*n* = 5) and docetaxel-treated (10 mg/kg weekly from W9 to W11, *n* = 5) PyMT mice were imaged. Parameters such as total vessel length, vascular density, diameter, and morphology of blood vessels were obtained by image analysis using ImageCell™ software (Mauna Kea Technologies) and automatic segmentation [10].

### *Ex Vivo* Analysis

#### Whole Mount 

In order to assess the tumor diffusion and detect the lymph node involvement, mammary glands were analyzed using the whole mount method. They were defatted, dehydrated, and stained with hematoxylin following the Rosen Laboratory *Mammary Gland Whole Mount Protocol* [11].

#### Histology 

Ki-67 and NIS staining were assessed on formalin-fixed paraffin-embedded tissues of normal and tumoral mammary glands, respectively, in FVB and PyMT mice, at W4, W6, W8, W10, W11, W12, and W14 (*n* = 2 for each time point) and in the treated groups. Mammary tumors were excised, fixed in 10 % paraformaldehyde in phosphate-buffered saline (PBS, pH 7.4), dehydrated, and embedded in paraffin. Serial sections were cut at 5 μm and stained with hematoxylin-eosin or processed for immunochemistry.

#### Immunohistochemistry 

Paraffin-embedded sections were deparaffinized and rehydrated through graded alcohols. Endogenous peroxidases were blocked with 3 % H_2_O_2_ during 5 min at 25 °C in Tris-buffered solution, washed, and processed for antigen retrieval with PBS (10 mM, pH 6) in a microwave oven (20 min, 750 W).

For Ki-67, sections were incubated with a goat anti-Ki-67 polyclonal IgG (Sc-7846, Santa Cruz Biotechnology Inc., Santa Cruz, USA) as primary antibody and a horse anti-goat IgG (PI-9500, AbCys) as secondary antibody, both at a concentration of 1:300 in 0.5 % Tween-PBS. For NIS, sections were incubated with a polyclonal rabbit anti-NIS at a concentration of 1:200. To reduce non-specific interactions of secondary antibodies, tissues were first incubated with 5 % normal serum from the host before incubation at 25 °C for 1 h with the primary antibodies. Endogenous peroxidases were blocked with 3 % H_2_O_2_ in water, and immunocomplexes were visualized by the ABC peroxidase method after staining with DAB for 2 min (Sigma-Aldrich, USA) and counterstained with hematoxylin-eosin. All histological slides were analyzed by an experienced pathologist (EM). In selected areas of hyperplasia, adenoma, and carcinoma localized in mammary glands using HES staining, the Ki-67 index was measured by eye-counting on 500–1,000 cells.

#### Tumor Growth Models 

Modelling of tumor growth was performed with Monolix 3.1 Release 2 (http://software.monolix.org/), a non-linear mixed effect model program that estimates both the structural model parameters as well as the between-subject (mice) and residual variability [12].

#### Statistical Analysis 

A nonparametric Mann–Whitney test was used for comparisons of MTV between groups: treated *vs.* untreated and doxorubicin- *vs.* docetaxel-treated. *P* values of 0.05 or less were considered statistically significant.

## Results

### Time Course of Tumor Progression and Detection by *In Vivo* Imaging

#### *Ex Vivo* Pathology

**Fig. 1:**
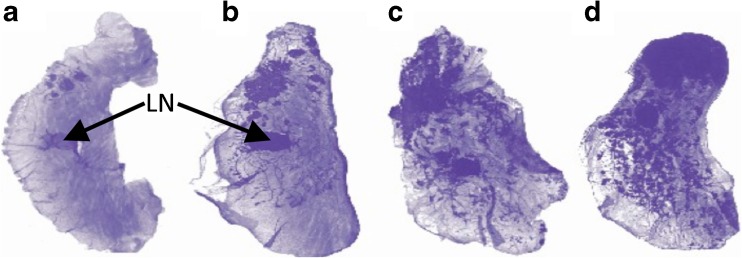
Spontaneous multifocal tumoral progression. Hematoxylin-stained whole mount sample of mammary glands from untreated PyMT mice at **a** W4, **b** W7, **c** W9, and **d** W10. *Arrows* point to the lymph node (*LN*) in the center of the mammary gland.

Whole mounts of excised tumoral mammary glands showed small foci of epithelial tumors as early as W4 (Fig. 1a). By W7–W8, all PyMT mice presented epithelial tumors of the mammary gland (Fig. 1b). Tumors appeared initially in the peri-areolar area and progressed throughout the mammary glands in a multifocal pattern along the ductal structures (Fig. 1a–c). Tumors extended readily and involved the whole gland by W10 (Fig. 1d).

#### CT and [^18^F]FDG Imaging

**Fig. 2:**
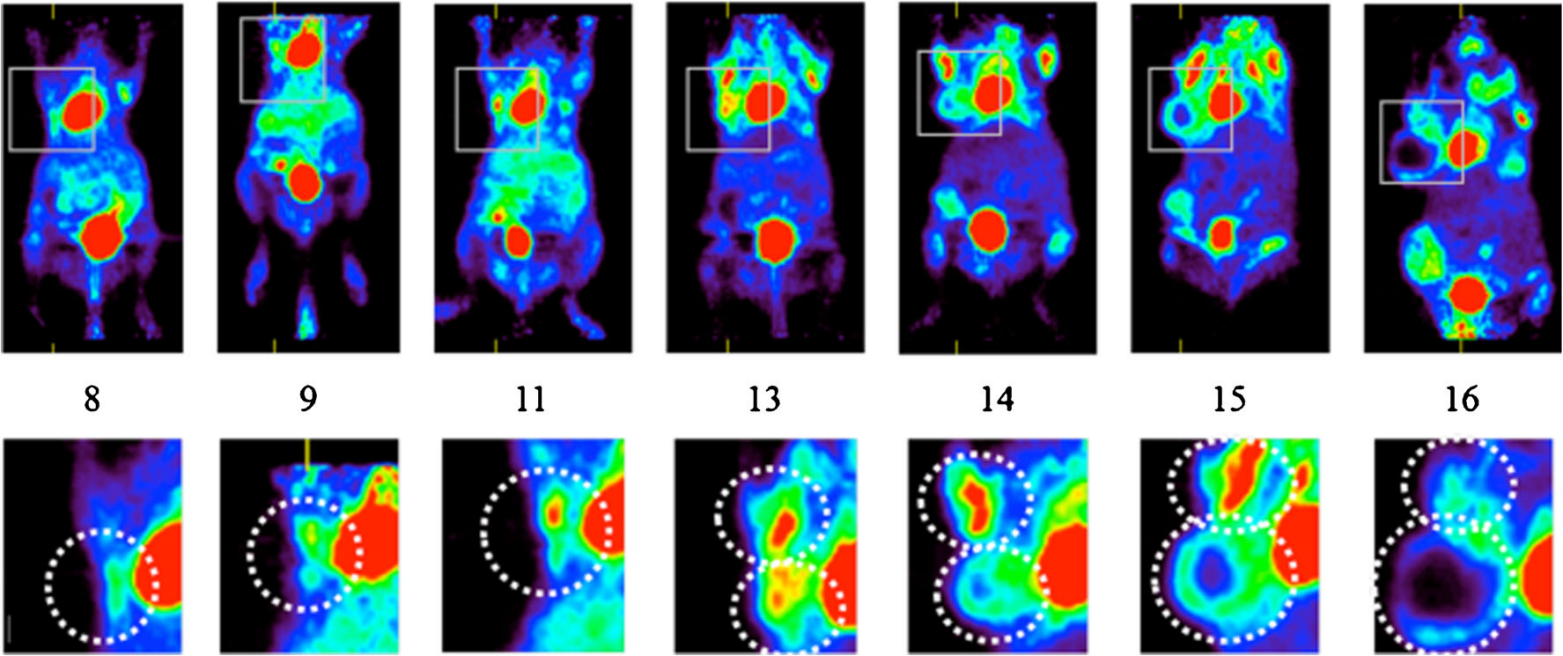
Longitudinal follow-up of tumor progression from W8 to W16 with [^18^F]FDG-PET. **a** Whole body coronal slices of a untreated PyMT mouse and **b** zoomed views of the thoracic mammary tumors (*numbers* indicate age in weeks). Note the hypometabolic central area indicating necrosis at W14–W16.

W8 corresponded to the earliest time point at which the tumors could be detected by palpation. CT images had a low tumor-to-background ratio and did not detect mammary tumors until they reached a size sufficient to modify the body contours of the mice (W14). In contrast, mammary tumors were detected on PET images as early as W8, as shown in a series of representative [^18^F]FDG-PET images acquired weekly from W8 to W16 in one mouse (Fig. 2). Mean MTV per mouse increased from 0.76 ± 0.59 cm^3^ at W9 to 7.45 ± 0.26 cm^3^ at W14, in a two-phase pattern: a slow growing phase from W8 to W11 followed by a fast growing phase thereafter. By W14, tumors had grown to large sizes and [^18^F]FDG-PET showed an hypometabolic central area (see the close up at W14 in Fig. 2) that *ex vivo* macroscopy confirmed to be necrosis. Lung metastasis could not be clearly identified *in vivo* by CT or PET, but a PET signal above background, possibly masking the uptake in small pulmonary lesions, was noted in the mediastinum, in addition to the myocardial uptake.

#### [^18^F]FLT Imaging and Comparison with Ki-67

**Fig. 3:**
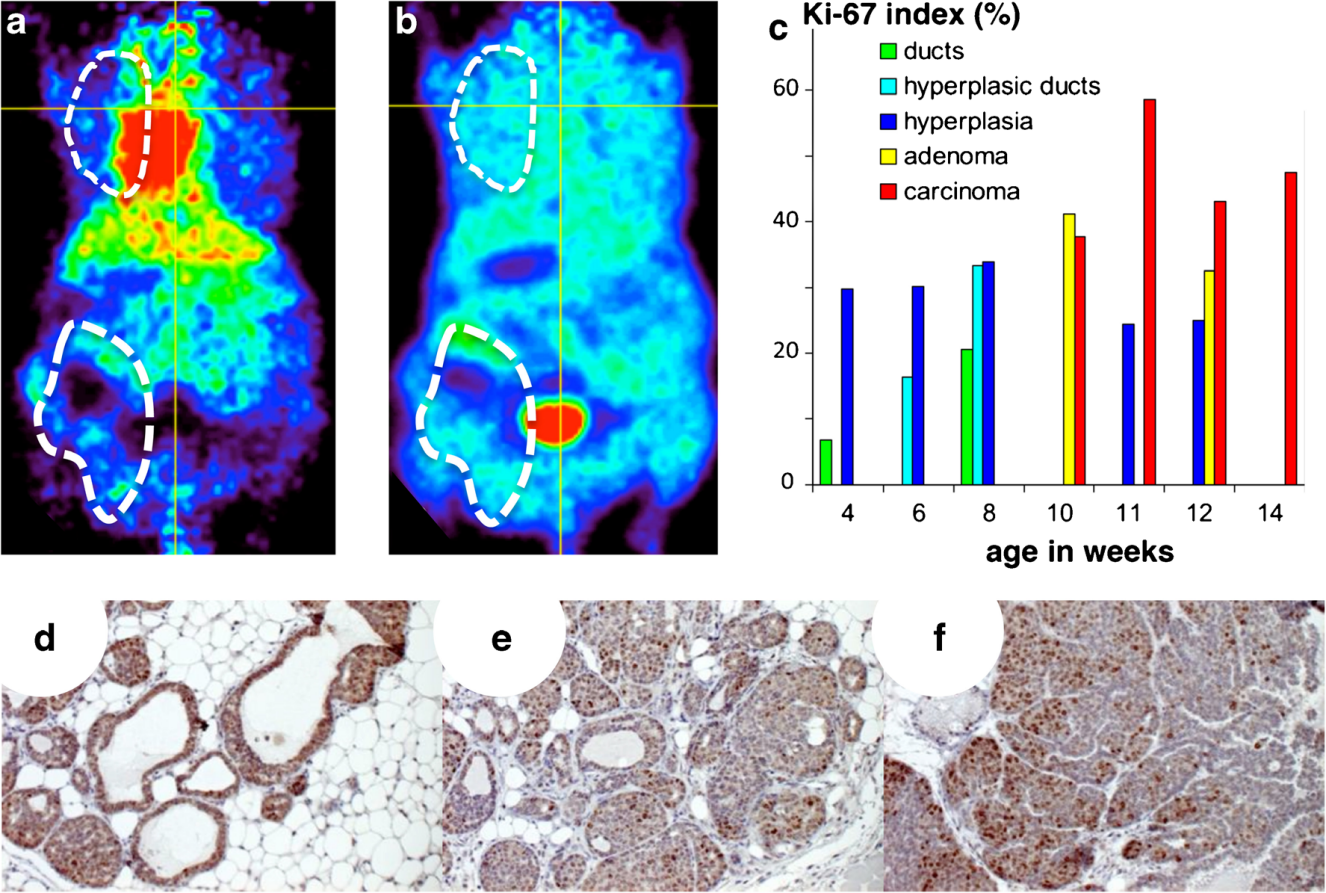
Coronal PET views acquired **a** 10 min and **b** 30 min after injection of [^18^F]FLT, showing the absence of tracer uptake in a PyMT mouse presenting large mammary tumors at W14. **c** Ki-67 index increases with tumor progression and is higher (35 % or more) in carcinoma areas than in adenoma and hyperplasic areas (mean from two PyMT mice at each time point). Increase in tumor cell density and proliferation demonstrated by Ki-67 staining of mammary glands in PyMT mice at **d** W10, **e** W12, and **f** W14. *Scale bars* 200 μm.

We found no contrast between the mammary tumors and the surrounding tissue of PyMT mice at 10 min (Fig. 3a) and 30 min (Fig. 3b) after [^18^F]FLT injection. Only background activity and tracer accumulation in the bladder were observed in images acquired at 30 min or at later times, up to 90 min after injection. Since [^18^F]FLT-PET is expected to reflect proliferation that is likely to be high during tumor development in the PyMT mice, we searched for the proliferation marker Ki-67 in serial histological sections of tumoral mammary glands of PyMT mice, at different stages of development.

Areas of hyperplasia, adenoma, and carcinoma were observed in mammary tumors, as previously described by Lin et al. [2]. Adenoma and carcinoma were present at W10 (Fig. 3c, d). The Ki-67 index was higher in carcinoma than in adenoma and hyperplasic areas (mean ± SD of 44 ± 15, 34 ± 12, and 25 ± 11 %, respectively) (Fig. 3c), and the cell density increased from W10 to W14 (Fig. 3d–f). The tumor proliferative activity assessed by the Ki-67 index was high at W14, with more than 40 % positive cells (Fig. 3c). Therefore, the absence of contrast on the [^18^F]FLT images cannot be attributed to the absence of proliferation in mammary tumors of PyMT mice. It is noteworthy that mammary glands of normal FVB mice showed at puberty (W4–W7) a median Ki-67 index of 6 ± 7 %, in agreement with ductal hyperplasia during pubertal mammary growth.

#### SPECT Imaging with [^99m^Tc]TEC

**Fig. 4:**
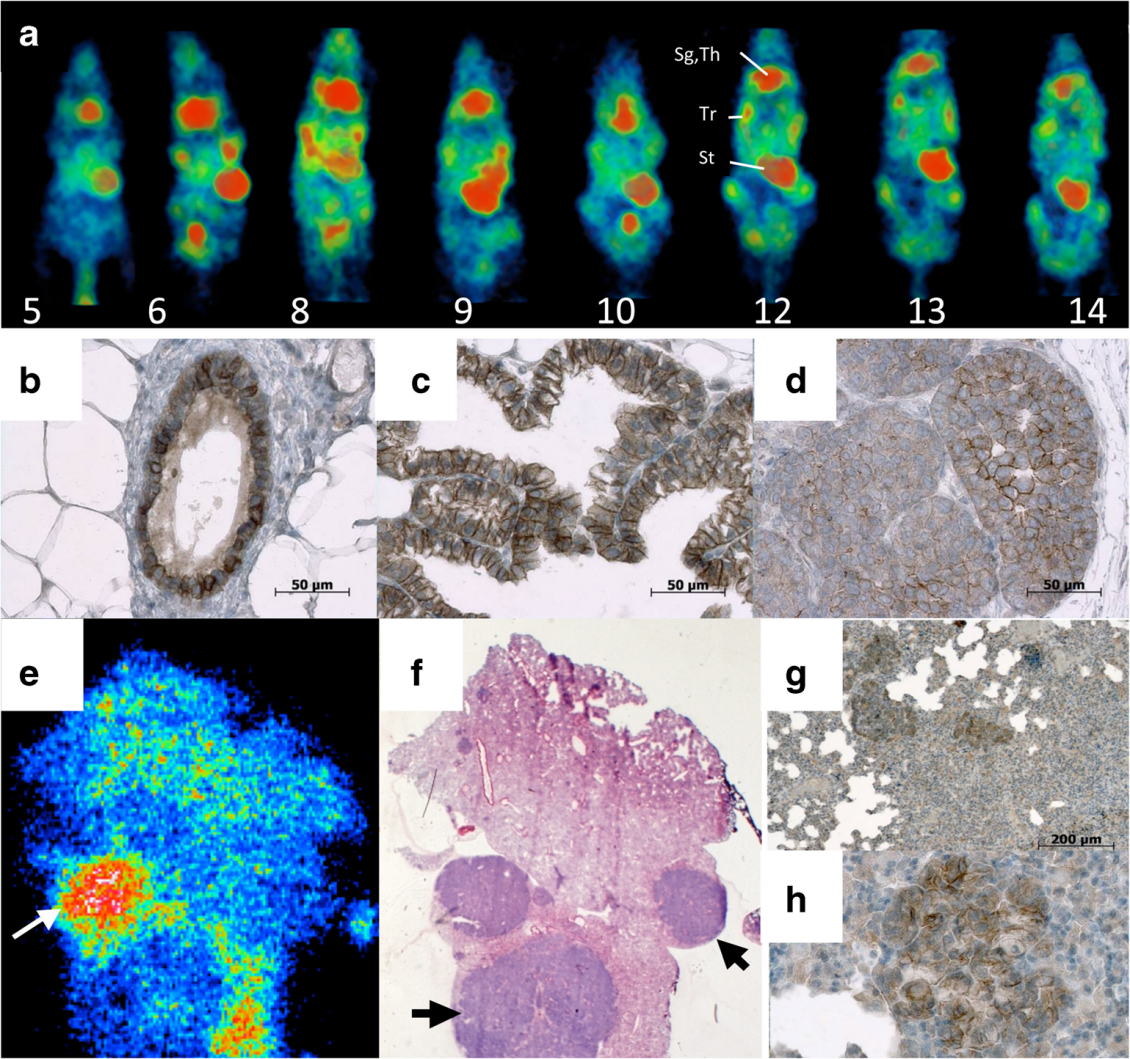
**a** Serial SPECT images after [^99m^Tc]TEC injection of a PyMT mouse from W5 to W14 (*numbers* indicate age in weeks). *Sg* salivary glands, *Th* thyroid gland, *Tr* mammary tumor, *Bl* bladder. Note [^99m^Tc]TEC uptake in the tumoral mammary glands already at W6. **b**–**d** Immunohistochemistry of NIS of the mammary gland in a FVB mouse at **b** W8 and in tumoral mammary glands of PyMT mice at **c** W8 and **d** W12; *scale bars* 50 μm. **e** *Ex* *vivo* autoradiography of lung in a PyMT mouse at W15 after administration of [^99m^Tc]TEC. [^99m^Tc]TEC is present in some lung metastases (*white arrow*) and **f** absent in other metastases staining positively for NIS as shown with *black arrows*. **g**, **h** Immunohistochemistry showing NIS labeling of the plasma membrane in lung metastases.

SPECT *imaging with [*^*99m*^*Tc]TEC* showed uptake in the mammary tumors of PyMT mice as early as W6 yielding clear contrast throughout to W14 (Fig. 4a). The mean TTV increased from 0.2 ± 0.2 to 7.5 ± 0.7 cm^3^ between W9 and W14. The tumor-to-background ratio was lower on [^99m^Tc]TEC SPECT images than on [^18^F]FDG-PET images. Staining of normal mammary glands for NIS showed an intense and ubiquitous labeling of the ducts (Fig. 4b), while in tumors, NIS was present in ductal as well as hyperplasic structures both on the plasma membrane and in the cytoplasm. NIS staining of tumoral tissue was more pronounced at W8 before treatment (Fig. 4c) than at W12 after treatment (Fig. 4d). Lung metastases were not detected on the SPECT images. To check whether this may have been the consequence of a low imaging contrast due to high background in the thoracic area (mediastinum and heart), we performed *ex vivo* autoradiography of lungs sampled after SPECT imaging. Interestingly, [^99m^Tc]TEC uptake was found in some lung metastases but absent in other metastases (Fig. 4e, f), although at the cellular level, NIS expression was observed in all lung metastases at a level similar to that found in primary mammary tumors (Fig. 4g, h).

#### *In Vivo* Fluorescence Imaging of Blood Vessels

With respect to normal mammary glands of FVB mice (Suppl. Fig. 1a), increased vascular densities and increased blood vessel diameters were clearly evident in the tumoral glands of PyMT mice injected with the vascular dye FITC-dextran and observed by FCE (Suppl. Fig. 1b). However, the parameters (mean vessel density, vessel length, and diameter) derived from the FCE images were widely variable, which precluded the use of FCE imaging as a quantitative tool for assessment of therapy efficacy in this model.

### Assessment of the Response to Chemotherapy

#### *Ex Vivo* Pathology and Histology

Histological whole mounts of mammary glands obtained from animals treated by weekly injections of 10 mg/kg doxorubicin at W9, W10, and W11 evidenced a clear regression of the lesions between W10 (Suppl. Fig. 2a) and W12 (Suppl. Fig. 2b) followed by recurrence at W14 (Suppl. Fig. 2c). In both doxorubicin- and docetaxel-treated groups, areas of tumor regression with nodular, scar, and fibrotic tissue and infiltration of inflammatory cells were observed at W10. Hyperplasic and carcinoma lesions resumed after discontinuation of chemotherapy, somewhat earlier in the docetaxel than in the doxorubicin group (W13 *vs.* W15). This difference might be explained by the shorter chemotherapy course for docetaxel than for doxorubicin (8 *vs.* 14 days), as suggested by the MTV growth curve showing earlier recurrence in the docetaxel group (see below).

#### [^18^F]FDG

**Fig. 5:**
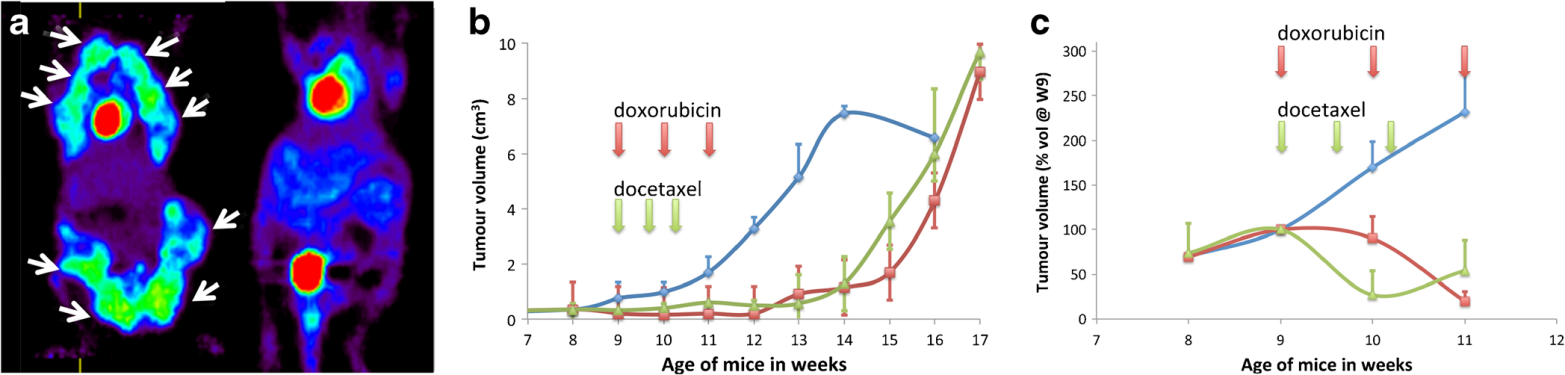
[^18^F]FDG-PET and [^99m^Tc]TEC-SPECT show reduced tracer uptake with chemotherapy. **a** Typical [^18^F]FDG-PET scan at W13 in an untreated (*left*) and a doxorubicin-treated (*right*) PyMT mouse. **b** Longitudinal assessment of the total metabolic tumor volume delineated by automatic segmentation from W8 to W14 in untreated (*blue curve*) and from W8 to W17 in doxorubicin- (*red curve*) and docetaxel-treated (*green curve*) PyMT mice (*n* = 4 for each group); tumor volumes are in cubic centimeter. Data points are mean ± SD of four independent measurements. **c** Same for [^99m^Tc]TEC uptake on SPECT images; tumor volumes are shown as percentage of the volume at W9.

Chemotherapy had a major effect on MTV measured with [^18^F]FDG in mammary tumors (Fig. 5a). In contrast to untreated animals in which MTV increased continuously to reach maximal values at W14, MTV in animals treated from W9 to W11 by doxorubicin or docetaxel remained at pretreatment levels over the duration of chemotherapy and until 3 weeks after its discontinuation until W13–W14 (Fig. 5b). Mean MTV was 0.18 ± 0.22 and 0.08 ± 0.16 cm^3^ at W9 and 0.86 ± 0.98 and 1.29 ± 1 cm^3^ at W14 in the doxorubicin- and docetaxel-treated groups, respectively (Fig. 5b). The change in MTV was statistically different between doxorubicin-treated group and untreated group (*p* = 0.028), docetaxel-treated group and untreated group (*p* = 0.03) and not between treated groups (*p* = 0.84).

After treatment discontinuation, MTV resumed its increase between W14 and time of euthanasia at W16-17, with a rate of increase in the treated group similar to the one measured in the untreated group between W11 and W14.

#### Tumor Growth Analysis

Different tumor growth models, including a Gompertz model, were tested to fit MTV derived from [^18^F]FDG-PET. Tumor growth was modeled as follows: MTV = MTV_0_ · *e*^kt^, where MTV_0_ is the extrapolated volume at time 0 (i.e., at W8 when tumors were first observed with PET), a proportional term to equilibrate the equation dimension and was fixed to 0.03 cm^3^ for the analysis, *k* is the time constant rate, *t* is the observation time in weeks, and the growth half-life is *T*_1/2_ = log(2) / *k*. The between-subject variability was modeled as an exponential error model and the residual variability as a combined (proportional plus constant) error model. The *k* estimates (% relative standard errors) were significantly different for the three groups: 0.315 (4 %), 0.145 (27 %), and 0.212 (36 %) week^−1^, yielding *T*_1/2_ of 2.2, 4.8, and 3.27 weeks in the untreated, doxorubicin, and docetaxel groups, respectively.

#### SPECT

At 1 week (W10) and 2 weeks (W11) after initiation of chemotherapy with docetaxel and doxorubicin, the TTV of [^99m^Tc]TEC uptake was unchanged or reduced with respect to pretreatment volumes. During the same time period, the TTV increased in untreated animals (Fig. 5c).

## Discussion

Clinically, 70–80 % of breast cancer patients respond to neoadjuvant chemotherapy, but a complete response is found in only 20–30 % [13–15]. Therefore, it is important to assess response to neoadjuvant drug therapy as early as possible in order to avoid unnecessary drug exposure. Functional and metabolic properties of tissues, including tumors, respond more rapidly than gross anatomy to changes in the environment; therefore, functional and molecular imaging modalities likely to monitor tumor response to treatment more precociously than tumor size are an important field in cancer research. Because they are based essentially on the same techniques than clinical imaging, small animal imaging devices are able to provide longitudinal follow-up in preclinical studies of new anticancer drugs [16]. Here, we compared the ability of several imaging methods to document the natural course of mammary tumor development in PyMT mice. We then tested the ability of standard imaging observables derived from these methods to document precociously, precisely, and predictively the response to chemotherapy.

### How Precocious Is Detection of Cancer by *In Vivo* Imaging in PyMT?

CT was able to detect the mammary tumors of the PyMT mice reliably only at W14–W15. CT is not well adapted to follow mammary carcinoma in mice because of a lower soft tissue contrast in small animals than in humans. In contrast, [^99m^Tc]TEC-SPECT and [^18^F]FDG-PET imaging showed a significant tumor-to-background contrast as early as W6 and W8, respectively (Fig. 4 or 2 and Suppl. Fig. 3), supporting the use of molecular imaging for early tumor detection in PyMT mice. The remarkably precocious contrast visible with [^99m^Tc]TEC-SPECT at W6 could be explained by the high overexpression of NIS in premalignant hyperplasic structures. Both [^99m^Tc]TEC and [^18^F]FDG were able to follow tumor progression in the PyMT model from early stages on, even though at later stages, [^99m^Tc]TEC uptake as well as NIS staining tended to decrease in advanced breast cancer lesions, in contrast to [^18^F]FDG measurements of MTV that increased until the end of the period of observation.

### How Precise Is Detection of Cancer by *In Vivo* Imaging in PyMT?

“Precise” imaging of cancer can mean one of two different things: (i) images that provide quantitative values of a cancer-specific molecular or functional hallmark or (ii) images that are proper surrogate markers of cancer progression.

Concerning the first aspect, in the present study, [^18^F]FDG-PET and [^99m^Tc]TEC-SPECT in PyMT mammary tumors showed increased metabolism and NIS expression, respectively. In contrast, [^18^F]FLT-PET did not reflect well the proliferation of tumor cells evidenced by Ki-67 labeling. Discrepancies between *in vivo* [^18^F]FLT uptake and proliferation have been reported in animal studies and explained by competition for [^18^F]FLT uptake by the high serum levels of thymidine in mice and the existence of a thymidine salvage pathway of variable importance between species and strains [17, 18].

Concerning the second aspect, LMA segmentation of PET dynamic images excluded the tumor regions with [^18^F]FDG concentrations not higher than background tissue. These regions often referred to as “necrotic” in animal studies were observed from W12 in the tumor cores of untreated mice and correspond to non-viable tumor tissue. Assessment of MTV with PET excludes these regions from the measurement of the tumor volume and improves the use of mathematical models of tumor growth and response to chemotherapy.

The size of the tumor began to increase exponentially starting at W10–11. FCE showed that this period coincides with a sudden change in the aspect of the tumor vessels and corresponds to the onset of the angiogenic switch [19], known to be associated with the progression towards carcinoma in this model [20].

### How Predictive of the Response to Chemotherapy Is *In Vivo* Imaging in PyMT?

#### [^18^F]FDG-PET

The effects of doxorubicin and docetaxel were assessed by measuring the metabolic tumor volume derived from *in vivo* images, each animal serving as its own control, a crucial point considering the inter-individual variability of tumor growth [5, 16]. Drugs were administered to PyMT mice at W9, 1 week after mammary tumors had become palpable. From this point of view, the present study reflects somewhat the clinical situation where imaging is challenged to report on the effect of drugs after diagnosis and before surgery.

Administration of two drugs commonly used in neoadjuvant breast cancer treatment [13–15] induced a prolonged delay of the increase in [^18^F]FDG uptake in the mammary tumors, at the expense of some toxicity. The *ex vivo* whole mount results combined with those of *in vivo* [^18^F]FDG-PET imaging support the view that MTV assessed using [^18^F]FDG were related to tumor regression and not to cellular stunning after administration of cytotoxic drugs. The difference between the doxorubicin group and the docetaxel group could be related to the nature of the drug, the dosage, or the duration of administration (14 *vs.* 8 days for doxorubicin and docetaxel, respectively).

Overall, our results support small animal [^18^F]FDG-PET for the assessment of response to chemotherapy, with the limitation that, as can be expected in a transgenic model in which tumorigenesis is genetically encoded, tumor progression resumes after chemotherapy discontinuation.

#### NIS-[^99m^Tc]TEC SPECT Imaging

NIS is a membrane glycoprotein of the follicular thyroid cells that actively transports iodine used for thyroid hormone biosynthesis. NIS was shown to be overexpressed in human breast cancers [21–23] and in several transgenic mice models including PyMT [21]. NIS overexpression was observed by immunohistochemistry in human breast cancers but not in normal tissue in a small series of samples [21] and confirmed in a larger series of 371 samples assessed by microarrays and histological techniques [22].

Surprisingly, in the present results, NIS overexpression was observed in normal mammary ducts in FVB mice, using three different immunohistochemical methods. This observation is not in agreement with the SPECT images showing [^99m^Tc]TEC uptake only in mammary tumors and not in normal mammary glands. Although NIS expression was more heterogeneous and at a lower level in mammary tumors than in normal breast ducts (Fig. 4), the higher cellular density in tumors than in normal mammary glands induced a higher contrast with the surrounding tissue. Although lung metastases are frequent in PyMT mice, confirmed by macroscopic analysis, the absence of [^99m^Tc]TEC uptake in some lung metastases on *ex vivo* autoradiography suggested the loss of NIS expression in metastatic lesions. Moreover, although radionuclide uptake has been shown to be correlated with NIS levels, cell surface NIS levels are variable in breast tumors [23].

#### The PyMT Transgenic Mouse as a Model to Study Mammary Cancer With *In Vivo* Imaging

Carcinogenesis in the PyMT mouse presents similarities with that of human breast cancer subtype with a multistep progression from an initial hyperplasic stage to invasive carcinoma [2]. Up to a certain extent, this animal model is relevant for dynamic studies of disease evolution and therapeutic intervention and its limitations are summarized in Table 1. A main difference with human disease is the occurrence of multiple points of tumor initiation at different stages ranging from hyperplasia to carcinoma. Cancer growth occurs during puberty in PyMT mice, and at advanced stages (W10 to W16), each mouse presents several tumoral foci on each mammary gland. However, from the imaging point of view, this drawback can be limited by the use of MTV and TTV to quantify [^18^F]FDG and [^99m^Tc]TEC, respectively.

**Table 1:** Strengths and weaknesses of the PyMT model

Strengths (≈ similarities with human disease)
Orthotopic (mammary gland) and *in situ* (ductal) tumors
Rapid: 2–3 months
Reproducible: 100 % penetrance in female homozygotes
Immune competent animals
Tumor development mimics human mammary carcinogenesis stages
Expresses many markers of breast cancer including hormone receptors
Lung and lymph node metastases
Weaknesses (≈ difference with human disease)
Genetic, not environmental etiology
Multifocal tumors with different stages
No brain or bone metastases
Juvenile disease

## Conclusion

*In vivo* molecular imaging of the PyMT mouse model of breast carcinoma by [^18^F]FDG-PET and [^99m^Tc]TEC SPECT is a precise tool to allow longitudinal monitoring and predict the tumor response to chemotherapy precociously.

## Electronic Supplementary Material

Below is the link to the electronic supplementary material.

ESM 1 (PDF 4,421 kb)
