# Supplementary material for: Multimodal In Vivo Imaging of Tumorigenesis and Response to Chemotherapy in a Transgenic Mouse Model of Mammary Cancer
Source: Mol Imaging Biol. 2015 Dec 2;18:617–26. doi: 10.1007/s11307-015-0916-7 (PMC4927598; doi:10.1007/s11307-015-0916-7)
Supplement: Supplementary file 1 — (PDF 4,421 kb) [file 11307_2015_916_MOESM1_ESM.pdf]

Electronic Supplementary Material

Multimodal In Vivo Imaging of Tumorigenesis and Response to Chemotherapy in a Transgenic Mouse Model of Mammary Cancer

Journal: Molecular Imaging and Biology

Jean-Louis Alberini* (1,2,3), Raphaël Boisgard* (1), Stéphanie Guillermet (1), Karine Siquier (1), Benoît Jego (1), Benoît Thézé (1), Saik Urien (4), Keyvan Rezaï (4), Emmanuelle Menet (5), Renaud Maroy (1), Frédéric Dollé (1), Bertrand Kühnast (1), Bertrand Tavitian (6,7)

1. CEA, DSV, I²BM, Service Hospitalier Frédéric Joliot, Laboratoire d'Imagerie Moléculaire Expérimentale, Orsay, France
2. Service de Médecine nucléaire, Institut Curie, Hôpital René Huguenin, Saint-Cloud, France
3. Faculté de Médecine, Université Versailles Saint-Quentin, France
4. Service de pharmacologie, Institut Curie, Hôpital René Huguenin, Saint-Cloud, France
5. Service de pathologie, Institut Curie, Hôpital René Huguenin, Saint-Cloud, France
6. Université Paris Descartes Sorbonne Paris Cité, Assistance Publique-Hôpitaux de Paris, Hôpital Européen Georges Pompidou, Radiology Department, Paris, France
7. Université Paris Descartes Sorbonne Paris Cité, INSERM UMR-S970, Cardiovascular Research Center - PARCC, Paris, France.

* These authors contributed equally.

Correspondence to Prof. Bertrand Tavitian, INSERM U970, PARCC, 56 rue Leblanc, 75015 Paris, France. Tel: +33 1 53 98 80 54, Fax: +33 1 53 98 79 52, bertrand.tavitian@inserm.fr

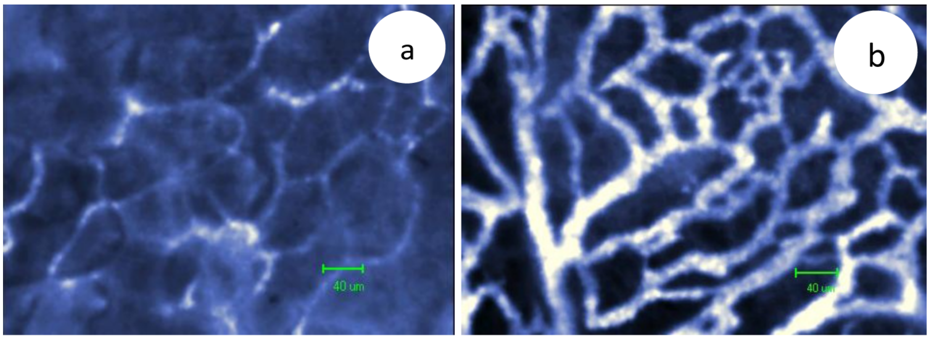

Suppl. Fig. 1 *In vivo* fluorescence imaging of blood vessels of a normal mammary gland in a FVB mouse at W12 (**a**) and of a mammary tumor in a PyMT mouse at W9 (**b**) using fluorescent confocal endomicroscopy (FCE). Scale bars 40 µm.

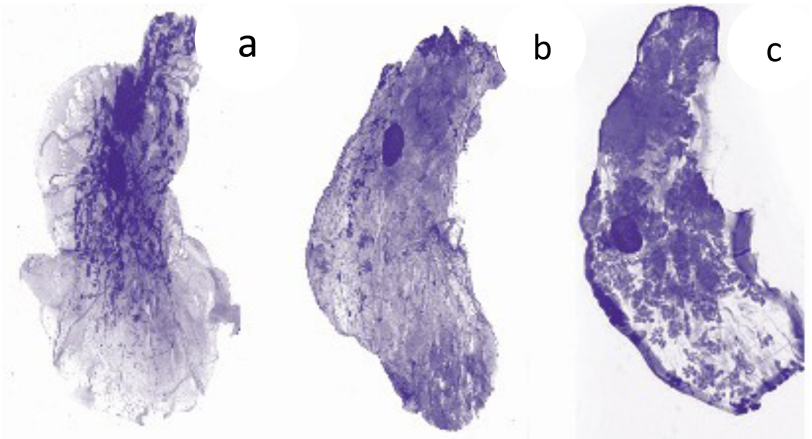

Suppl. Fig. 2 Whole mount of mammary glands of PyMT mice at W9 before treatment (**a**) showing tumor regression under doxorubicin at W12 (**b**) and tumor recurrence after discontinuation of treatment at W14 (**c**).
